# Cascadedness in Chinese written word production

**DOI:** 10.3389/fpsyg.2015.01271

**Published:** 2015-08-25

**Authors:** Qingqing Qu, Markus F. Damian

**Affiliations:** ^1^Key Laboratory of Behavioral Science, Institute of Psychology, Chinese Academy of SciencesBeijing, China; ^2^School of Experimental Psychology, University of BristolBristol, UK

**Keywords:** handwriting, written production, orthography, cascadedness, lexical access, Chinese

## Abstract

In written word production, is activation transmitted from lexical-semantic selection to orthographic encoding in a serial or cascaded fashion? Very few previous studies have addressed this issue, and the existing evidence comes from languages with alphabetic orthographic systems. We report a study in which Chinese participants were presented with colored line drawings of objects and were instructed to write the name of the color while attempting to ignore the object. Significant priming was found when on a trial, the written response shared an orthographic radical with the written name of the object. This finding constitutes clear evidence that task-irrelevant lexical codes activate their corresponding orthographic representation, and hence suggests that activation flows in a cascaded fashion within the written production system. Additionally, the results speak to how the time interval between processing of target and distractor dimensions affects and modulates the emergence of orthographic facilitation effects.

## Introduction

Interactivity has been highlighted as a central principle in current thinking about mental representations and processes involved in language (e.g., Boland and Cutler, 1996; Rapp and Goldrick, 2000). The term interactivity refers to the possibility that multiple cognitive processes influence one another as they take place. Models of spoken language production describe the way in which thought is transformed into spoken output, a process which involves access to semantic-syntactic and phonological encoding levels. At the former level, a lexical target node is selected among a cohort of activated semantically related lexical nodes, while at the latter level phonological codes of words are accessed, and an issue which has occupied theorists for a long time is how activation is transmitted between the two levels. According to a “serial” view of language production represented by the model of Levelt et al. (1999), lexical-semantic access and phonological encoding represent separate and discrete processing steps: semantic-syntactic processing ends once the target lexical node is selected, and subsequent phonological encoding is restricted to the selected target node. Other lexical candidates which might be co-activated in response to conceptual process do not activate their corresponding phonological codes. By contrast, an interactive view of spoken production has been advocated by Dell and O'Seaghdha (1991, 1992): according to this view, semantic-syntactic and phonological processing take place simultaneously, and form properties of targets can impact on lexical retrieval. A critical component of the claim that word production is “non-discrete” is represented by the assumption that activation transmission from semantic to phonological levels is “cascaded.” Cascadedness implies that (a) access to phonology begins before semantic-syntactic retrieval has been completed, (b) all candidates which are activated at the semantic level, and not just the target, are allowed to influence phonological encoding (see Rapp and Goldrick, 2000, for an extensive analysis).

The question of whether or not multiple phonological codes are activated in word production has been extensively studied within the spoken word production literature. Although as of yet no full consensus on this issue has emerged, earlier evidence appeared to support the serial position (e.g., Schriefers et al., 1990; Levelt et al., 1991) but a number of studies have over the last few years reported evidence which shows multiple phonological activation and hence supports the view that activation flows in a cascaded fashion (e.g., Peterson and Savoy, 1998; Morsella and Miozzo, 2002; Navarrete and Costa, 2005; Meyer and Damian, 2007; Oppermann et al., 2008, 2010; Görges et al., 2013).

Compared to spoken production, relatively less research has been carried out to investigate the processes and mechanisms underlying written word production, and very little work has been devoted to investigate whether activation flow is serial or cascaded when a word is written (see e.g., Bonin and Fayol, 2000; Roux and Bonin, 2012). Partially this lack of evidence can be attributed to the fact that in alphabetic languages such as English, orthography and phonology are heavily confounded, hence it is not easy to construct studies which could disentangle the two variables. Because in non-alphabetic scripts such as Chinese, orthography, and phonology are largely dissociated, their corresponding effects can be better isolated from each other. The work reported below investigated how activation flows between lexical selection and orthographic encoding in Chinese written production, and specifically sought to address whether lexical access in written word production takes place in a cascaded or serial manner. We will begin by reviewing the main empirical evidence on cascadedness in spoken production, and then outline the limited evidence that addresses the issue in written production before introducing our experiment.

### Seriality vs. cascadedness in spoken word production

Whether word production is characterized by serial or by cascaded processing can be investigated with the following research strategy. Two stimuli (or two dimensions of a single stimulus) are presented simultaneously, phonological relatedness between them is manipulated, and it is investigated whether responses to the target stimulus (or dimension) are affected. If so, this would imply that participants involuntarily phonologically encoded not only the target, but also the non-target dimension. Such a finding would be at odds with the serial view, but compatible with the cascaded notion. For instance, Peterson and Savoy (1998) asked participants to name a series of objects which were selected to have two possible names (near-synonyms; e.g., couch-sofa), with the dominant (couch) and subordinate (sofa) names established in a pilot study. On occasional critical trials, speakers were cued not to name the object, but rather to name a word which was presented immediately following the object. The word was either phonologically related to the dominant object name (count-couch), phonologically related to the subordinate object name (soda-sofa), or unrelated. Relative to the unrelated condition, word naming was faster when the word was phonologically related to the dominant picture name. Crucially, facilitation was also found when the word was related to the subordinate picture name. This finding suggests that phonological representations corresponding to both names of the object were activated, which is not predicted by a serial account but is consistent with the cascaded view according to which a target activates multiple word forms. However, it is possible that synonyms represent somewhat atypical instances in that multiple names exist for near-identical conceptual representations. Hence cascading in spoken word production might be restricted to such cases of extreme competition in which two lemma nodes are activated to an equal level (Levelt et al., 1999).

More recently, Morsella and Miozzo (2002) introduced a task (henceforth called the “picture-picture priming” paradigm) which provides clearer evidence for cascadedness. Two superimposed pictures were presented in two different colors, and speakers were instructed to name the target object (e.g., shown in green) while attempting to ignore the distractor object (e.g., shown in red). Target and distractor objects were typically unrelated, but on some trials they were phonologically related (e.g., bed-bell). Latencies were faster on trials with related than with unrelated object names, which suggests that not only the target but also the distractor picture was phonologically encoded, as expected from the cascaded view. Similar results were subsequently obtained in Spanish by Navarrete and Costa (2005) and in English by Meyer and Damian (2007); however, note that Jescheniak et al. (2009) failed to obtain picture-picture priming in German. In a variant of this task, Roelofs (2008) measured not only vocal response latencies, but also eye movements. Superimposed pictures were presented on the left side of the screen and participants named the target; subsequently on each trial they were asked to press a key in response to arrows pointing left or right, presented on the right side of the screen. This allows to measure gaze shift latencies as the time interval between the beginning of the first fixation and the end of the last fixation for the picture naming task. It was found that gaze shift latencies were shorter in the phonologically related condition relative to the unrelated condition, which again indicates that phonological activation is not confined to the target picture but extends to the distractor picture, in line with the cascaded view.

In the picture-picture priming task, the visual display which results from two objects being superimposed onto each other can at times be perceptually quite complex. Hence, perhaps speakers might on occasion mistakenly select the distractor picture rather than the target picture first, and start to process the target picture only when they notice the mistake. Hence, the typical priming effect obtained when target and distractor name are related may come about due to a problem with the selection of targets (Navarrete and Costa, 2005). To reduce the chances of failure to select the target picture, Navarrete and Costa introduced what we will refer to as a “picture-color priming” task in which target and the distractor dimension are easier to discriminate at the physical level. In this task, a colored object is presented, and speakers are asked to report the name of the color while ignoring the picture. Navarrete and Costa manipulated the phonological relationship between the picture and color name (e.g., vela verde, “green candle” vs. roca verde, “green rock”; the study was conducted with Spanish speakers). The result showed faster color naming in the related than the unrelated condition, which suggests that the phonological properties of pictures were activated even when speakers were instructed to ignore them (see Janssen et al., 2008; Kuipers and La Heij, 2009; Dumay and Damian, 2011, for parallel evidence from English, French, and Dutch color naming, respectively). Results from the picture-picture, the picture-color priming, and other tasks hence converge on the inference that non-target dimensions are phonologically encoded. This suggests that in spoken word production, activation is transmitted from lexical selection to phonological encoding in a cascaded fashion (see also Blanken et al., 2002, for a relevant aphasic case study, and (Bles and Jansma, 2008), for neuroimaging results).

### Seriality vs. cascadedness in written production

Compared to spoken production, relatively less work has been devoted to investigate the processes and mechanisms underlying written production. It is generally assumed that written and spoken production involve similar high levels of processing, e.g., conceptual retrieval and lexical-semantic selection (Caramazza and Hillis, 1990; Van Galen, 1991; Bonin et al., 1998; Bonin and Fayol, 2000) and subsequently diverge into phonological encoding for spoken utterances, and graphemic encoding for written ones (see Perret and Laganaro, 2012, for an EEG study highlighting this assumption). Further, evidence from neuropsychological patients suggests that orthographic codes can be accessed directly from semantics (“orthographic autonomy”; e.g., Rapp et al., 1997) rather than orthographic access being necessarily mediated by phonological codes (e.g., Geschwind, 1969). At the same time, there is mounting evidence for a role of phonology in orthographic encoding (see e.g., Bonin et al., 2001; Zhang and Damian, 2010; Qu et al., 2011). Hence, orthographic output codes are likely accessed both from a direct semantic route, and from an indirect phonological pathway.

With regard to the transmission route from semantic retrieval to orthographic encoding, the same question as in the spoken domain arises: is activation transmitted in a serial or a cascaded fashion? To our knowledge, only two studies provide evidence to address this question. Bonin and Fayol (2000) employed a picture-word interference task in which French participants wrote responses on a graphic tablet. They found effects of semantic and phonological target-distractor relatedness; crucially, when targets and distractors were both semantically and phonologically related (e.g., cheval-chien, “horse-dog”) an interaction was obtained such that the semantic effect was eliminated in the simultaneous presence of phonological relatedness. A similar interaction had been previously documented with spoken responses (Starreveld and La Heij, 1995; Damian and Martin, 1999; Taylor and Burke, 2002). Based on Sternberg's (1969) additive-factors logic, Bonin and Fayol argued that as semantic and phonological relatedness affect separate processing stages, the interaction arises because the underlying processing stages interact, hence supporting a cascaded/interactive processing view. Whether this inference is valid is not clear, however. At present a controversy exists concerning the locus of semantic effects in PWI tasks (e.g., Mahon et al., 2007; Abdel Rahman and Melinger, 2009). Semantic effects in PWI task were conventionally interpreted as evidence for competition between co-activated lexical entries (e.g., Glaser and Glaser, 1982; Schriefers et al., 1990; Roelofs, 1992; Starreveld and La Heij, 1995; Levelt et al., 1999). However, this interpretation has recently been challenged by the “response exclusion hypothesis” according to which semantic effects in the PWI task arise at a postlexical, articulatory stage (see Mulatti and Coltheart, 2012; Spalek et al., 2012; for recent overviews of this controversy). To the extent to which this debate remains unresolved, it is difficult to interpret the empirical interaction between semantic and phonological relatedness found in PWI tasks. An additional problem specific to the interaction in the written domain shown by Bonin and Fayol (2000) is that form-related distractors were not only phonologically, but also orthographically related to the picture names. This confound makes it difficult to identify whether activation cascades from semantics to orthographic, or to phonological, representations, given the mounting evidence alluded to earlier that written word production is constrained by phonological activation.

More direct support for a cascaded view of written word production comes from a recent picture-picture priming study reported by Roux and Bonin (2012). Adopted from the task introduced by Morsella and Miozzo (2002) two pictures were superimposed on each other in two colors, and French participants were asked to write the name of the target (green) picture while attempting to ignore the distractor (red) picture. It was found that phonologically and orthographically related distractor pictures (bougie-banc; “candle-bench”) facilitated the writing latencies of target pictures. More importantly, this kind of facilitation effect was also found when target and distractor picture names shared the initial grapheme but started with a different phoneme (cigare-camion, “cigar-truck”); by contrast, no effect was found when target and distractor shared the initial phoneme but not the initial grapheme (souris-citron, “mouse-lemon”). These findings offer support for the view that activation cascades from the semantic level to the stage of orthographic encoding.

### The current study

The study reported below pursues a similar approach and aimed at supplying further evidence concerning the issue of whether written production is cascaded or serial. We sought to extend on the work by Roux and Bonin (2012) in the following ways. First, we investigated written production in Chinese individuals, using an orthographic system which is not alphabetically organized. Given the considerable dissimilarity between alphabetic systems used in Western languages and ideographic systems such as Chinese orthography, it needs to be investigated whether processing characteristics shown in one system (in this case, cascadedness in French written word production) generalize to other systems. An additional benefit of using Chinese as the target language is that in such non-alphabetic systems, orthographic, and phonological dimensions are largely dissociated from one another, which allows for a clear manipulation of form overlap in an experimental design. And because (as outlined above) phonology almost certainly constrains written word production, it is particularly important to ensure that observed effects reflect the semantics-orthography transmission route, rather than an indirect route via phonology. In Chinese it is straightforward to select word pairs which, although phonologically unrelated, share a substantial portion of their orthographic properties (in the study below, a radical within a character). Finally, in the picture-picture priming task employed by Roux and Bonin, a potential problem is that superimposing two line drawings of objects creates displays which can vary dramatically in visual complexity. This could introduce differences between experimental conditions which potentially distort the results. Morsella and Miozzo (2002) addressed this issue with a control experiment in which the same picture combinations were named by speakers of a different language in which the picture pairs were phonologically unrelated. They interpreted the null finding in this control experiment to imply that displays were matched across conditions concerning visual complexity and other non-linguistic variables. Roux and Bonin also reported two control experiments, one of which consisted of a manual name-picture matching task which is typically assumed to capture perceptual and conceptual, but not lexical components of picture processing (Stadthagen-Gonzalez et al., 2009; but see Chu and Meyer, 2010, for problems with this inference) and one involving a natural/man-made decision on the target object. Based on a null finding in both tasks they argued that differential visual complexity between conditions is unlikely to have influenced the results. However, it would be best to avoid this potential problem altogether, and so we employed a written version of the picture-color priming task introduced by Navarrete and Costa (2005) in which a colored object is presented on each trial, and participants respond with the color and try to ignore the object. In this task, target (color) and distractor (object) dimensions are integrated, hence no issues concerning visual complexity arise.

Moreover, we manipulated the stimulus onset asynchrony (SOA) interval between the color (target) and picture (distractor) dimension. A colored object was either displayed as such (both dimensions are available simultaneously; SOA = 0 ms), or the object was first shown in black lines for a very brief period (−300 or −150 ms) before its lines were colored, hence making the distracting dimension available slightly ahead of time. In production tasks of this type, SOA is often manipulated to gain insight into the time course of an effect (or pattern of effects; e.g., Schriefers et al., 1990; Damian and Martin, 1999). In the present study, the motivation was mainly to minimize the likelihood of missing crucial evidence regarding the central issue of serial vs. cascaded information flow. Under any SOA, if written production of the color name is affected by orthographic overlap with the picture name, this would suggest that not only the target but also the to-be-ignored object name were orthographically encoded, that is, orthographic activation occurs for task-irrelevant candidates. Hence, obtaining an orthographic priming effect would contravene one of the key assumptions of the serial view (activation of word forms is restricted to the target) but support a cascaded view of orthographic word production (multiple word forms are activated).

## Method

### Participants

Thirty native Mandarin Chinese speakers (all writers of simplified Chinese characters, as is predominantly the case in the mainland of China), most of them students from Beijing Forest University and China Agricultural University and the minority students at the University of Bristol, were paid for their participation. All participants had normal or corrected-to-normal vision and no history of dysgraphia. None of the participants were color blind. This study was approved by the Ethics Committee of the University of Bristol and Institute of Psychology, Chinese Academy of Sciences. Written informed consent was obtained from all participants.

### Materials and design

Four colors (orange, green, brown, and blue) were used as target responses to be written, and 12 objects from the Snodgrass and Vanderwart (1980) picture set were chosen as the distractors (three per color). All four color names were monosyllabic in Chinese and hence are written as a single character, and all picture names were disyllabic hence consisting of two orthographic characters. None of the selected objects had a canonical color (“yellow banana”).

The critical manipulation was that for the “orthographically related” condition, each of the 12 objects was combined with a color such that the color name shared a radical with the first character of the object name (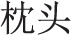, pillow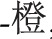, orange). For the “unrelated” condition, colors and objects were combined such that no orthographic overlap existed (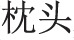, pillow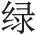, green). Hence, 24 critical combinations (12 related, 12 unrelated) were formed. Semantic relationship between pictures and colors was avoided, and specifically, there was no phonological overlap in either the related or the unrelated condition. A complete list of experimental materials is presented in Table A1. To reduce the likelihood that participants may notice the relationship between picture and color and predict the color from the object name, 12 filler pictures were added in order to reduce the percentage of related trials. As was the case for the critical target pictures, each filler picture was paired with two colors, thus forming 24 filler trials in which pictures and colors had no semantic, orthographic, and phonological overlap.

Picture-color SOA was manipulated as −300, −150, and 0 ms. At SOA = −300 and −150 ms, an object was first presented in black lines for 300/150 ms, and then immediately replaced by the same object in colored lines. At SOA = 0 ms, a colored object was presented throughout, hence both dimensions were available simultaneously. Trials were blocked by SOA; the order of SOA blocks for each participant was determined by a Latin square design. Under each SOA, all 24 critical trials (12 related, and 12 unrelated) and the 24 filler trials were presented. In this way, each SOA block consisted of 48 trials, and the entire experimental session of 144 trials, out of which 36 (25%) were orthographically related. A new pseudorandom order was generated for each block and participant. Neither pictures nor colors were repeated on consecutive trials.

### Apparatus

The experiment was run using DMDX (Forster and Forster, 2003) from a personal computer on a 19-in. monitor. Response latencies, i.e., the interval between target (color) onset and initial contact of the pen with the tablet, were recorded by a WACOM Intuos A4 graphic tablet and a WACOM black inking pen. Participants wrote their responses on an A4 sheet of paper attached to the tablet. Objects were standardized to a size of approximately 5 × 5 cm and were presented at the bottom of the screen in order to reduce head and eye movements between the screen and the writing surface.

### Procedure

Participants were tested individually in a quiet room. They first were asked to familiarize themselves with the experimental stimuli by viewing all 24 pictures presented in reduced size and their names on the computer screen. This was mainly done because for a few of the stimuli, naming agreement was less than optimal (but note that Navarrete and Costa, 2005, found a cascadedness effect in a color-picture priming task regardless of whether participants had been familiarized with the objects or not; see also Kuipers and La Heij, 2009, who asked participants in separate halves of their experiment to either name the object or the color, and found significant facilitation even when color naming took place in the first half, i.e., objects had not been previously named). Participants were then instructed that their task would be to write down, on each trial, the color in which an object was presented. In a subsequent practice block, eight pictures with colors which were not related to the object names were presented. Each of the four target colors was presented twice. Participants were asked to write down the color name as fast as possible while attempting to ignore the pictures. They were instructed not to lift the pen too far away from the answering sheet so that initiation of the response would not require an arm movement; neither should they drop the pen on the sheet before knowing what word they would write. Compliance with these instructions was assured before the experiment began. The experimenter corrected wrong responses (e.g., incorrect color naming or wrong color character writing) in the practice phase. Then, the three experimental blocks of 48 trials each were carried out, separated by short breaks. Each testing session lasted approximately 25 min.

On each trial, participants saw a sequence consisting of a fixation cross (500 ms), a blank screen (500 ms), a picture, and an inter-trial interval (1000 ms). As described above, at SOA = 0 ms, a colored picture was shown, whereas at SOA = −300 and −150 ms, a picture was first shown in black lines, and then after 300 or 150 ms replaced with the same picture in color. Colored pictures remained on the screen for 3000 ms, and responses were collected during this period. Subsequently, the stimulus was removed and the next trial began.

## Results

Only responses on critical trials were analyzed. Response latencies were discarded from the analysis if the response consisted of the incorrect color or character (3.2%) or when a latency was faster than 200 ms or longer than 2500 ms (0.6%). Mean reaction latencies, error percentages, and facilitation effects for each condition are shown in Table 1. Given the limited number of items (four colors) we did not perform item analysis (see Spinks et al., 2000; Navarrete and Costa, 2005, Experiment 2 for the same strategy).

**Table 1:** **Mean response latencies (RT, in milliseconds) and mean error percentages (PE)**.

**Condition**	**SOA**						**Overall**	
	**−300 ms**		**−150 ms**		**0 ms**			
	**RT**	**PE**	**RT**	**PE**	**RT**	**PE**	**RT**	**PE**
Orthographically related	766	1.4	772	1.9	792	1.4	777	1.6
Unrelated	805	4.7	802	4.4	794	5.6	800	4.9
Effect	+39^**^	+3.3^*^	+30^**^	+2.5^*^	+2	+4.2^**^	+23^**^	+3.3^**^

SOA, Stimulus-Onset Asynchrony.

** p < 0.01;

* p < 0.05.

**Figure 1:**
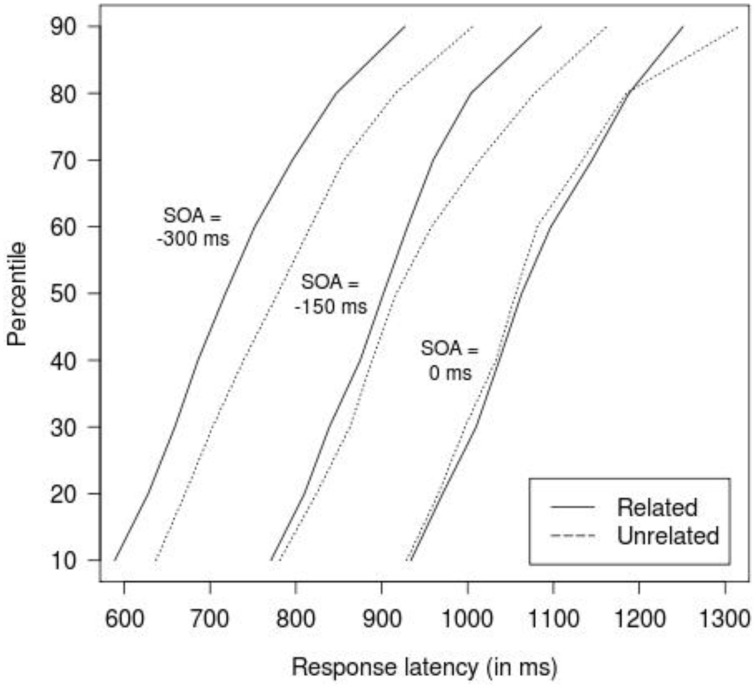
**Mean cumulative response latency distributions, dependent on relatedness (related vs. unrelated), and SOA (−300, −150, 0 ms)**. To improve legibility, curves for SOA = −150 ms have been shifted 150 ms to the right, and curves for SOA = 0 ms have been shifted 300 ms to the right.

An analysis of variance (ANOVA) was conducted on response latencies that included relatedness (related vs. unrelated) and SOA (−300, −150, 0 ms) as within-participants variables. The results revealed a highly significant main effect of relatedness, *F*_(1, 29)_ = 9.50, *MSE* = 32, 294, *p* = 0.004, showing that response latencies were faster in the related condition than in the unrelated condition. The main effect of SOA was not significant, *F* < 1. The interaction between relatedness and SOA was significant, *F*_(2, 58)_ = 3.17, *MSE* = 6757, *p* = 0.049. Tests that assessed the effects of relatedness at each SOA separately showed significant facilitation at SOA = −300 ms, *t*_(29)_ = 2.85, *p* = 0.008, and SOA = −150 ms, *t*_(29)_ = 2.93, *p* = 0.007, but not at SOA = 0 ms, *t*_(29)_ < 1, *p* = 0.848. Figure 1 shows cumulative frequency distributions of latencies, calculated separately for each participant and decile and then averaged, indicating that the effect at SOA = −300 and −150 ms extends across the entire latency range.

A parallel analysis conducted on the errors yielded a significant main effect of relatedness, *F*_(1, 29)_ = 8.771, *MSE* = 500, *p* = 0.006, with participants making fewer errors in the related condition than in the unrelated condition. Neither SOA nor the interaction between SOA and relatedness was significant, *Fs* < 1. Table 2 presents an analysis of the writing errors by error type and SOA. As can be seen, the majority of errors (64%) was classified as semantically as well as orthographically related to the correct target (i.e., responses which consisted of an incorrect color name which shared one or more character constituents with the target color, such as 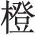, /cheng2/, “orange” → 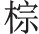, /zong1/, “brown”). Purely orthographic errors were less common (20%) and only a single purely semantic error, in the absence of orthographic relatedness to the target, was observed. Error types appeared reasonably well distributed across the three SOAs.

**Table 2:** **Types of writing errors, sorted by overall frequency of error type**.

**Error type**	**SOA**			**Overall**
	**−300 ms**	**−150 ms**	**0 ms**	
Semantically and orthographically similar	18 (82)	11 (48)	16 (64)	45 (64)
Orthographic	3 (14)	7 (30)	4 (16)	14 (20)
No response	1 (5)	4 (17)	1 (4)	6 (9)
Recording error	0	1 (4)	2 (8)	3 (4)
Semantic	0	0	1 (4)	1 (1)
Ambiguous response	0	0	1 (4)	1 (1)
Total	22	23	25	70

Numbers are raw occurrences; numbers in parentheses represent percentages of errors under a given stimulus-onset asynchrony (SOA).

## Discussion

In the present study, we investigated the issue of how information flows within the written word production system. Using a task in which native Mandarin speakers were presented with colored objects and wrote down the color while attempting to ignore the object, we found a reliably significant facilitation effect on response latencies when color and object name shared an orthographic radical (23 ms overall), and we showed a gradient of orthographic priming dependent on color-object SOA such that the effect was significant at SOA = −300 ms (39 ms) and at SOA = −150 ms (30 ms), and not significant at SOA = 0 ms (2 ms). It should be noted that with regard to errors we also found overall significant priming (3.3%), and orthographic facilitation under all three SOAs. Stimuli for our study had been chosen such that the only relationship between the color and picture was orthographic, whereas phonological or semantic overlap was avoided. In this way, we were able to dissociate orthographic from phonological relatedness and obtain a “pure” orthographic priming effect. Moreover, the same objects and colors were used in the related and unrelated condition, ruling out the possibility that stimuli differed across the two conditions. In order to discourage potential strategies from participants (i.e., predicting the color name from the object name in the related condition) we added filler pictures to reduce the percentage of related trials (25% in the study). Additionally, we asked participants after each testing session if they had noticed a relationship between picture and color names; none of them reported that they had noticed any overlap. The presence of an orthographically based priming effect from the irrelevant picture names onto written color naming constitutes clear evidence that non-target lexical nodes activate their corresponding orthographic representation. This finding suggests that activation flows in a cascaded fashion within the written word production system.

With regard to their theoretical implications, our results are compatible with those recently reported by Roux and Bonin (2012) with French participants. To reiterate (see Introduction), these authors used a picture-picture priming task in which the two objects were sometimes form-related, and documented a facilitatory effect which was based on orthographic overlap. Our study extends their findings to the Chinese written word production system, and by demonstrating purely orthographic facilitation effects it highlights cascadedness as a fundamental property of written production. In other words, despite the dramatic differences between the French and Chinese orthographic systems, the underlying processing characteristics appear to be similar.

Apart from speaking to the general principles of activation flow in written production, the results also provide some insight into the unique nature of orthographic representations of non-alphabetic scripts such as Chinese. Our central finding is that an overlapping orthographic radical between a to-be-named color name and to-be-ignored object name induces a facilitation effect. The Chinese orthographic system can be described at different levels, i.e., strokes, radicals, characters, and words, and it is common to assume that there is a sublexical representational level for radicals. Results from a number of studies support this assumption. For instance, Zhou and Marslen-Wilson (1999) asked Chinese participants to name target words, and obtained a priming effect when prime words were semantically related to phonetic radicals of the target words, even though prime words were semantically unrelated to the whole target words. Radical processing in Chinese writing has also been indicated by few neuropsychological studies. Law (1994, 2004) and Law and Caramazza (1995) analyzed writing errors made by a group of Cantonese dysgraphic patients and observed numerous errors at the radical level (i.e., radical replacement, deletion or insertion) which also support the claim that radicals form important mental representations. The fact that our own results show orthographic priming from to-be-ignored object names which shared a radical with the color names further underscores the psychological reality of the representational level of radicals in Chinese individuals.

On methodological grounds, we believe that our picture-color priming task is preferable to Roux and Bonin's (2012) picture-picture priming, for the reasons outlined in the Introduction: because target (color) and distractor (object) dimension are integrated, no potential confounds from perceptual complexity can arise which potentially afflict the object combinations in the picture-picture priming task and necessitate complex control experiments to rule them out. No such control experiments were necessary in our experiment.

Our results provide some insight into how the time interval between processing of target and distractor dimensions affects and modulates the emergence of orthographic facilitation effect. In the latencies, a gradient was found such that orthographic facilitation was more pronounced at the earlier SOAs, and absent at an SOA of 0 ms. This pattern likely arises from the relative time course of processing of the two dimensions (object and color): in our study, color names were monosyllabic in Chinese and hence are written as a single character, and all picture names were disyllabic hence consisting of two orthographic characters. Therefore, it is quite plausible that in our study, orthographic encoding of distractors (object) is slower than that of targets (color). Evidently, with simultaneous presentation (at SOA = 0 ms), activation cascading from the object arrives at the orthographic level too late to have an effect on orthographic encoding of the color word. By contrast, when the object dimension is processed slightly ahead of time (at negative SOAs) activation cascading from the object to orthography arrives just in time to facilitate color writing.

The observation that in our results priming was strongest at negative SOAs but was present at SOA = 0 ms only in the errors poses an interesting contrast with Roux and Bonin's (2012) study of written word production in which only a single SOA (0 ms) was used and priming was found in latencies. Similarly, in parallel studies on spoken production using the color naming task (Navarrete and Costa, 2005; Kuipers and La Heij, 2009; Dumay and Damian, 2011) substantial priming was found with simultaneous presentation of colors and objects. In tasks with two dimensions such as picture-picture and picture-color priming, the outcome depends on the time course of target and distractor processing, as well as on the exact timing of the two dimensions relative to each other (i.e., SOA). In our own experiment, target dimension processing could have been faster, and/or distractor processing slower, for priming to emerge at an “earlier” SOA than in Roux and Bonin's study. Overall, it is difficult to attach significance to particular SOA patterns when comparing across tasks, stimulus sets, and response languages. Nevertheless, it is important to note that, had we included only the typical SOA of 0 ms, we may have erroneously concluded (based on our null finding at response latencies at that SOA) that Chinese written word production is not cascaded. This underscores the importance of including a range of SOAs in studies of this type to minimize the odds of obtaining false negatives.

One might propose an alternative position of the SOA effect, that is, the SOA manipulation may generate two sequential serial processes for object and color, respectively. The scenario would be such that, at negative SOAs in which objects are presented before colors, participants may automatically process the objects and thus activate the corresponding orthographic code. Subsequently, information about the color is made available, and due to the awareness of task requirements, participants start to process the colors, suppress information about the distractor (object names in this case), and eventually produce the color name. In this scenario, both the target (color in this case) and distractor (object) lexical nodes are selected successively and thus orthographic codes for both dimensions are activated. In this case, an orthographic effect would be compatible with a serial view. Indeed, a similar interpretation of the effect as a result of misselection has been proposed by Bloem et al. (2004) to query the phonological picture-picture effect observed in Morsella and Miozzo (2002). Bloem et al. argued that this phonological effect might be due to an erroneous selection of the distractor object on some of trials, followed by a covert suppression of the object name and eventual production of the target object name. This hypothesis was evaluated by Roelofs (2008) by examining the latency distributions of responses in the related and unrelated conditions, based on the assumption that if the effect reflected genuine cascading of activation, then it should be present through the entire latency range; by contrast, if it resulted from misselection, then it should be present in slow responses only. This analysis showed the effect to be present through the entire latency range and increasing linearly with latency, hence discrediting the misselection hypothesis. In the present study, we likewise found that the orthographic effect was present across the latency distribution range (see Figure 1) and take this as evidence that the effect results from genuine cascading.

A further argument against the alternative scenario arises from the prediction that, if participants first covertly named the object and then switched to color naming, the properties of objects and their names should influence color naming latencies. As a tentative test of this prediction, we performed a correlational analysis between object name frequency and response latencies and found no correlation. Finally, we argue that the alternative account of our effect would in fact predict an *inhibitory* effect of relatedness on color naming latencies, rather than the observed facilitatory effect, because object names would need to be suppressed before color writing can proceed, which should be more difficult in the related than in the unrelated case. Relevant evidence comes from the observation that bilinguals find it harder to carry out spoken color naming in their second language when color and object are phonologically related in their first language (Macizo, 2015), which is predicted on the claim that co-activated representations which are primed via phonological overlap in L1 are more difficult to suppressed than unrelated representations. Having said that, we acknowledge that our case for cascadedness (rather than the alternative account in terms of “multiple serial” processing sketched above) would be stronger if orthographic priming had been found not only at strongly “negative” SOAs (in which information about object identify precedes arrival of information about the target color) but also under shorter SOAs (ideally, under SOA = 0 ms). It has to be kept in mind that we found significant priming in the errors at SOA = 0 ms.

Our results, suggesting cascadedness to the orthographic level in Chinese written word production, contrast in an interesting way with very recent results from Chinese spoken word production reported by Zhu et al. (2015). In a picture-word interference tasks in which semantic and form overlap are factorially crossed, the two variables typically interact with each other (e.g., Starreveld and La Heij, 1995), a pattern which has been taken to imply non-seriality in spoken word production. By contrast, Zhu et al. showed with Chinese speakers (a) additive effects of semantic and phonological overlap, (b) via an analysis of electroencephalography, a strictly serial sequence of semantic followed by phonological access. The authors concluded that “temporal signatures associated with spoken word production might differ depending on target language” (p. 16). If this inference is correct, then the interesting issue arises why their results with spoken responses suggested seriality, whereas ours with written responses suggested cascadedness (and hence imply non-seriality). This issue cannot easily be resolved without additional evidence, perhaps from an EEG-based study of Chinese written word production in which semantic and form overlap is factorially crossed. At minimum, the contrast between the two sets of findings cautions against prematurely generalizing from one task or paradigm to another.

In summary, a number of previous studies addressing spoken word production have recently provided evidence for a cascaded information processing view: selection of a target word activates multiple candidates at the phonological level. The present study demonstrated that the cascadedness principle is not merely limited to spoken word production but that it also applies to written production, with multiple orthographic forms being activated by a target. On a broader level, our inferences converge with results from the dysgraphia literature which had been taken to suggest cascadedness. Sage and Ellis (2004) reported an individual who was impaired at the level of the graphemic buffer as evidenced by exhibiting letter errors, length effects, and few lexical errors, and who displayed a significant effect of lexical variables such as word frequency and age of acquisition on spelling accuracy. Buchwald and Rapp (2009) demonstrated similar effects of lexical properties on letter accuracy in individuals with impairment in the graphemic buffer. Buchwald and Falconer (2014) examined writing-to-dictation performance of a patient with dysgraphia resulting in frequent semantic substitutions as well as errors at the letter level, suggesting that both lexical and letter level processing were impaired in this individual. Crucially, letter accuracy was lower for words with weaker activation during lexical access than for words with stronger activation, suggesting a cascaded transmission principle between the two representational levels.

In the present study, we focused on “central” processing stages in writing and found evidence for cascadedness between semantic and orthographic levels. A separate but related issue which has recently received some interest is the relation between “central” and “peripheral” stages in handwriting. For instance, Kandel and colleagues have reported a series of empirical studies in which the motor output of written production was analyzed in detail, and “central” variables such as orthographic regularity, and syllabic, graphemic, and morphological structure were shown to emerge in motoric characteristics such as inter-letter intervals (e.g., Kandel et al., 2006, 2011, 2012; Kandel and Spinelli, 2010; Roux et al., 2013). Results from these and related findings suggest that activation cascades from central to peripheral processing levels in writing. A functional interaction between central planning and peripheral motor stages was also found during learning how to write in young children of ages 8–10 year-old (Kandel and Valdois, 2005; Kandel and Perret, 2015). However, our knowledge concerning this issue comes largely from studies conducted with alphabetic languages and healthy adults; by contrast, our understanding of non-alphabetic languages and children and adults with dyslexia remains quite limited. Further research will be needed to investigate these issues. Overall, however, the currently available results highlight cascadedness as a central principle of processing in word production.

## Author note

This work was supported by the Scientific Foundation of Institute of Psychology, Chinese Academy of Sciences, under Grant No. Y3CX132005, and the National Natural Science Foundation of China, No. 31400967, to the first author.

### Conflict of interest statement

The authors declare that the research was conducted in the absence of any commercial or financial relationships that could be construed as a potential conflict of interest.

## Appendix

**Table A1:** **Stimulus materials used in the experiment**.

**Target color**	**Related picture**	**Unrelated picture**
(orange, cheng2)	(cup, bei1zi)	(peanut, hua1sheng1)
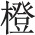
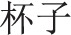
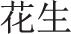
(orange, cheng2)	(comb, shu1zi)	(vase, hua1ping2)
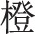
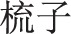
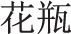
(orange, cheng2)	(chair, yi3zi)	(spinning wheel, fang3che1)
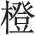
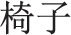
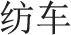
(green, lü4)	(cotton reel, xian4zhou2)	(cup, bei1zi)
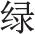
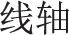
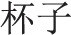
(green, lü4)	(sheep, mian2yang2)	(pillow, zhen3tou2)
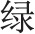
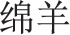
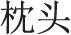
(green, lü4)	(spinning wheel, fang3che1)	(squirrel, song1shu3)
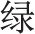
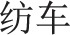
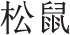
(blue, lan2)	(peanut, hua1sheng1)	(rubber, xiang4pi2)
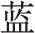
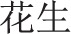
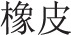
(blue, lan2)	(vase, hua1ping2)	(comb, shu1zi)
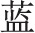
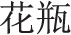
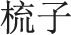
(blue, lan2)	(fly, cang1ying)	(chair, yi3zi)
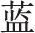
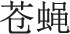
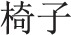
(brown, zong1)	(rubber, xiang4pi2)	(cotton reel, xian4zhou2)
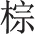
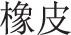
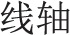
(brown, zong1)	(pillow, zhen3tou2)	(sheep, mian2yang2)
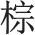
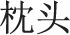
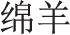
(brown, zong1)	(squirrel, song1shu3)	(fly, cang1ying)
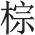
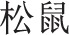
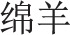

Numbers in the transcription of Chinese words represent tones (neutral tone is not marked).
